# Supplementary material for: Universal Josephson diode effect
Source: Sci Adv. 2022 Jun 8;8(23):eabo0309. doi: 10.1126/sciadv.abo0309 (PMC9176746; doi:10.1126/sciadv.abo0309)
Supplement: 20220608-1 [file sciadv.abo0309.v1.pdf]

CONDENSED MATTER PHYSICS

Universal Josephson diode effect

Margarita Davydova*, Saranesh Prembabu, Liang Fu*

Copyright © 2022 The Authors, some rights reserved; exclusive licensee American Association for the Advancement of Science. No claim to original U.S. Government Works. Distributed under a Creative Commons Attribution License 4.0 (CC BY).

We propose a universal mechanism for the Josephson diode effect in short Josephson junctions. The proposed mechanism is due to finite Cooper pair momentum and is a manifestation of simultaneous breaking of inversion and time-reversal symmetries. The diode efficiency is up to 40%, which corresponds to an asymmetry between the critical currents in opposite directions $I_{c+}/I_{c-} \approx 230\%$. We show that this arises from both the Doppler shift of the Andreev bound state energies and the phase-independent asymmetric current from the continuum. Last, we propose a simple scheme for achieving finite-momentum pairing, which does not rely on spin-orbit coupling and thus greatly expands existing platforms for the observation of supercurrent diode effects.

INTRODUCTION

Recently, there has been a surge of interest in nonreciprocal phenomena in superconductors. In particular, recent experiments have observed an asymmetry between forward and reverse critical currents $I_{c+} \neq I_{c-}$ in superconducting films (*1*–*3*) and Josephson junctions (*4*–*8*). In the presence of such a nonreciprocity, currents of magnitudes in the range between I_{c+} and I_{c-} can flow without resistance in only one direction, resulting in supercurrent diode effect. The dissipationless superconducting diodes should be contrasted with the asymmetric current-voltage characteristics of conventional semiconductor diodes, which are based on resistive and dissipative transport.

The supercurrent diode effect can occur when the free energy is asymmetric under the sign change of the supercurrent. This requires time-reversal symmetry breaking, which can be achieved by an external magnetic field or an exchange field from magnetic proximity effect. As an example, in the presence of Zeeman field, a noncentrosymmetric superconductor with spin-orbit interaction can acquire finite Cooper pair momentum, resulting in the so-called helical superconducting state (*9*, *10*). Recent theory predicts (*11*–*15*) that these systems exhibit supercurrent diode effect, where the critical currents along and against the direction of the Cooper pair momentum have different magnitudes. Thus, the supercurrent diode effect is a direct manifestation of unconventional superconductivity with broken time-reversal and inversion symmetries.

Supercurrent nonreciprocity has also been observed in a number of experiments on Josephson junctions (*4*–*8*, *16*), often referred to as Josephson diode effect (JDE). While several theoretical proposals based on various physical mechanisms have been put forward (*17*–*26*), a clear understanding of the microscopic origin of the JDE observed in experiments is still lacking. Very recently, an experiment (*7*) showed simultaneous occurrence of the JDE and finite Cooper pair momentum in a superconductor-normal-superconductor junction where two niobium electrodes were coupled by a thin flake of topological semimetal $NiTe_2$. Moreover, the observed features of the JDE, such as the temperature and the magnetic field dependence of $\Delta I_c \equiv I_{c+} - I_{c-}$, were accounted for by a phenomenological model based on finite-momentum Cooper pairing (*7*). This suggests a previously unknown link between the JDE and finite-momentum Cooper pairing. However, a microscopic theory relating these two phenomena is yet to be developed.

In this work, we present a universal theory of JDE due to Cooper pair momentum in short Josephson junctions. An analytical formula for the Josephson current is obtained for a short junction between finite-momentum superconductors, which generalizes the well-known result for zero-momentum superconductors. We find an asymmetry between the critical currents in opposite directions that is directly related to the Cooper pair momentum. We propose a simple scheme for achieving finite-momentum pairing and thus inducing the JDE, which is based on the Meissner effect. This scheme only involves a small magnetic field $H < H_{c1}$, does not require spin-orbit coupling, and is applicable to all superconductors. Thus, our work greatly expands the material platform for observing the JDE.

Our mechanism of the JDE has the following origin. The presence of finite Cooper pair momentum results in Doppler energy shift of quasiparticle energies by $\pm q v_F$ for left and right movers with momentum close to $\pm k_F$, respectively. Because left and right movers carry currents in opposite directions, by breaking their degeneracy the Doppler shift causes the Josephson current to be direction dependent and thus gives rise to the JDE, as we show below. We find that the contribution from the continuum of states plays important role for determining the magnitude of the asymmetry between the critical currents in opposite directions.

Unlike previous proposals, the JDE that we found in short junctions is universal: It is independent of the junction parameters and occurs already in the ballistic limit. It arises from and provides a measure of the Doppler shift of quasiparticle energy due to Cooper pair momentum. The proposed mechanism does not rely on scattering between multiple conduction channels (*17*–*21*), layered magnetic structures (*23*), curved geometry of a nanowire (*24*), or doped Mott insulator region at the interface (*25*). We find that the diode effect remains present in the presence of disorder in the system and potential barrier in the junction.

RESULTS

Finite-momentum pairing from the Meissner effect

We consider a short weak link between two superconducting regions as shown in Fig. 1, where both superconductors have Cooper pair momentum q. Thus, the superconducting order parameter (pair potential) in regions 1 and 2 is

$$\Delta_1(x) = \Delta e^{2iqx},\ \Delta_2(x) = \Delta e^{i\varphi + 2iqx} \quad (1)$$

Department of Physics, Massachusetts Institute of Technology, Cambridge, MA 02139, USA.
*Corresponding author. Email: luthien@mit.edu (M.D.); liangfu@mit.edu (L.F.)

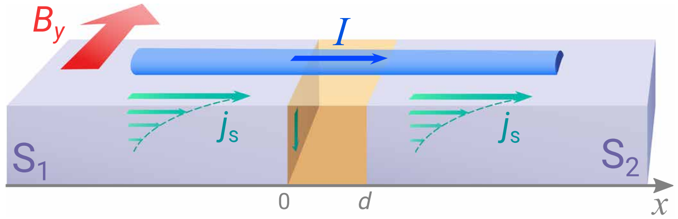

Fig. 1. Schematic illustration of a short Josephson junction. A metallic nanowire bridge is placed on top of two superconducting slabs. Because of an applied in-plane magnetic field $B_y < B_{c1}$, screening currents j_s emerge in the superconductors leading to finite-momentum Cooper pairing and periodically modulated pairing potentials ($\Delta_{1,2} \sim e^{2iqx}$) at the surface of the slabs S_1 and S_2. Consequently, the proximity-induced pairing potential in the metallic nanowire acquires the same spatial modulation.

respectively, where φ is the overall phase difference between them. Note that $q \neq 0$ breaks both inversion and time-reversal symmetry, which is necessary for the JDE. Our goal is to calculate the current-phase relation for this setup and demonstrate the asymmetry between the critical currents in opposite directions.

While finite-momentum superconductivity is thought to be elusive, the finite-momentum Josephson junction—the subject of our study—can be achieved by placing a normal metal bridge or semiconductor wire on top of two conventional superconductors, as shown in Fig. 1. Applying a small in-plane magnetic field $B_y < B_{c1}$ in the direction perpendicular to the wire induces a screening current on the surface of each superconductor. Correspondingly, the superconducting order parameter on the surface develops a spatially modulated phase $\theta(x) = qx$, where q is the Cooper pair momentum (assuming the gauge $A = 0$ within the superconductor). For thick superconducting slabs, $q \approx B_y\lambda_L$ where λ_L is the London penetration depth.

By means of superconducting proximity effect, the induced pair potential in regions 1 and 2 of the normal metal or semiconductor bridge (see Fig. 1) inherits the spatially modulated phase $\theta(x)$ from the superconducting order parameters on the surface of the two superconductors. Our setup thus realizes the required condition (1) for finite-momentum Josephson junction. Here, the proximity-induced gap Δ is generally smaller than the gap of the parent superconductor. The Cooper pair momentum q is controlled by the external magnetic field. Note that this scheme for creating finite-momentum pairing does not rely on spin-orbit coupling but instead uses surface screening current that is universally present in the Meissner phase of all superconductors. A clear evidence of finite-momentum pairing has recently been observed in thin Bi_2Te_3 layers proximitized by the superconductor $NbSe_2$ under a small magnetic field on the order of 10 mT (*27*). We also discuss alternative ways to achieve finite Cooper pair momentum later.

The current-phase relation

We find the current-phase relation for a short junction with two superconducting regions at $x < 0$ and $x > d$, where the junction length is much smaller than the induced coherence length $d \ll \xi_{\text{ind}} \propto \frac{v_F}{\Delta}$. The order parameters in the superconducting regions are given in Eq. 1, and in the normal region $0 < x < d$, we assume $\Delta(x) = 0$. Without loss of generality, we assume that $\Delta > 0$. In what follows, we solve for $N = 1$ mode in the junction. It is straightforward to generalize this approach to the case of multiple modes.

The BdG Hamiltonian in the superconducting regions is

$$\mathcal{H} = \int dx (\psi_\uparrow^\dagger, \psi_\downarrow) \begin{pmatrix} -\frac{\partial_x^2}{2m} - \mu & \Delta(x) \\ \Delta^*(x) & \frac{\partial_x^2}{2m} + \mu \end{pmatrix} \begin{pmatrix} \psi_\uparrow \\ \psi_\downarrow^\dagger \end{pmatrix} \quad (2)$$

where Δ(*x*) in left and right superconducting regions are defined in Eq. 1. Assuming that the chemical potential is large $\mu \gg \Delta$, we linearize the kinetic energy near momenta $\pm k_F$, which correspond to right and left movers, respectively. We also rotate the wave functions in the superconducting regions 1 and 2 according to $e^{i(qx+\varphi_{1,2}/2)\tau_z}$, where $\varphi_1 = 0$, $\varphi_2 = \varphi$, and τ_z is the Pauli matrix in the Nambu basis. Thus, we arrive at an especially simple Hamiltonian

$$\begin{aligned} \mathcal{H} &= \frac{1}{2}\int dx \left[(c_{\uparrow+}^\dagger, c_{\downarrow-}) H_+ \begin{pmatrix} c_{\uparrow+} \\ c_{\downarrow-}^\dagger \end{pmatrix} + (c_{\uparrow-}^\dagger, c_{\downarrow+}) H_- \begin{pmatrix} c_{\uparrow-} \\ c_{\downarrow+}^\dagger \end{pmatrix} \right] \\ H_+ &= \begin{pmatrix} v_F(-i\partial_x + q) & \Delta \\ \Delta & -v_F(-i\partial_x - q) \end{pmatrix}, \\ H_- &= \begin{pmatrix} -v_F(-i\partial_x + q) & \Delta \\ \Delta & v_F(-i\partial_x - q) \end{pmatrix} \end{aligned} \quad (3)$$

where the notation "+/−" pertains to the states with momentum near $\pm k_F$, respectively. Note that in an infinite system described by this Hamiltonian, due to the Doppler effect from Cooper pair momentum, $\Delta \pm qv_F$, the quasiparticle dispersion has two spectral gaps for quasiparticles moving in the right and left directions, correspondingly.

We use the scattering matrix formalism developed in (*28*, *29*) to find the spectrum of the bound states. In the absence of normal reflection, it is given by roots of the equation

$$\left((r_A^+)^2 - e^{2iqd+i\varphi} \right) \left((r_A^-)^2 - e^{-2iqd-i\varphi} \right) = 0 \quad (4)$$

The effect of the normal reflection is discussed later in the text. Here, $r_A^\pm$ are up to a phase proportional to the Andreev reflection amplitudes at the interface for right- and left-moving particles

$$r_A^\pm = \frac{E \mp v_F q}{\Delta} - i\sqrt{1 - \left(\frac{E \mp v_F q}{\Delta}\right)^2} \quad (5)$$

The presence of two different coefficients $r_A^\pm$ is a direct consequence of the broken time-reversal and inversion symmetries.

In the absence of any normal reflection, the right- and left-moving states are decoupled and produce two separate bound states

$$\begin{aligned} E_1 &= -\Delta\cos\frac{\tilde{\varphi}}{2} + v_F q, \; -\Delta + qv_F < E < \Delta + qv_F \\ E_2 &= \Delta\cos\frac{\tilde{\varphi}}{2} - v_F q, \; -\Delta - qv_F < E < \Delta - qv_F \end{aligned} \quad (6)$$

where we introduced the notation $\tilde{\varphi} = \varphi + 2qd$. The constant phase $2qd$ is negligible in short junctions and is not relevant to the discussion of the supercurrent nonreciprocity.

The spectrum is shown in Fig. 2; for $\tilde{\varphi}$ near 0 and 2π, the branches enter the continuum and the resulting spectrum is 2π periodic in $\tilde{\varphi}$. The bound state energies generalize the known result for the short junctions at $q = 0$ (*28*, *30*). In the presence of finite Cooper pair momentum $q \neq 0$, the key difference is that the branches for right- and left-moving particles are shifted in energy by $\pm qv_F$.

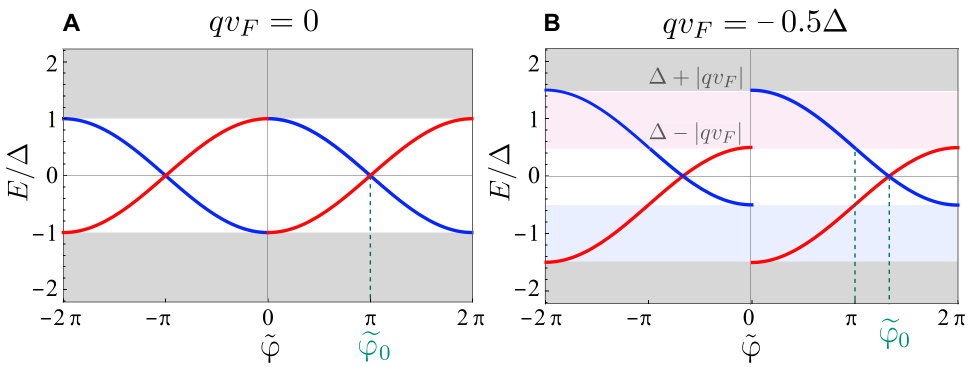

Fig. 2. Spectra of the bound states in a perfectly transparent junction. (**A**) Zero and (**B**) nonzero Cooper pair momentum $2q$. The bound states originating form left- and right-moving states are shown in blue and red, respectively.

The Josephson current can be determined as $I = \frac{2e}{\hbar}\frac{dF}{d\varphi}$, where F is the free energy of the system (*29*). At zero temperature, the current is

$$I = -\frac{2e}{\hbar}\sum_{E>0}\frac{dE}{d\varphi} - \frac{2e}{\hbar}\int_{E\in\text{cont.}}^{\infty} dE\, E\frac{d\nu(E)}{d\varphi} \quad (7)$$

The first term is the contribution from the bound states, and the second term corresponds to the current from the continuum of states; here, $\nu(E)$ is the density of states in the continuum. Thus, we decompose the total current as $I = I_{\text{bound}} + I_{\text{cont}}$. The contribution from the bound states equals

$$I_{\text{bound}} = -\frac{2e}{\hbar}\frac{d\,|\,E_{\text{bound}}\,|}{d\varphi} = \frac{e\Delta}{\hbar}\sin\frac{\tilde{\varphi}}{2}\,\text{sgn}\left(\Delta\cos\frac{\tilde{\varphi}}{2} - qv_F\right) \quad (8)$$

Unlike in conventional short Josephson junctions, the branch change does not occur at π anymore but is determined by the zero of argument of sgn(. .) function, namely, $\tilde{\varphi}_0 = 2\arccos\left(\frac{qv_F}{\Delta}\right)$, which is shown in Fig. 2B. This is the consequence of the shift of the two bound state energies seen Fig. 2B.

Let us now discuss the second contribution to the Josephson current. When time-reversal symmetry is broken, the continuum of states is known to contribute to the Josephson current [see (*24*, *31*) and the discussion in the Supplementary Materials]. The density of states $\nu(E)$ in the junction can be evaluated as (*29*)

$$\nu(E) = -\frac{1}{\pi}\text{Im}\frac{\partial}{\partial E}\ln\det\,(1 - s_A s_N) + \text{const.} \quad (9)$$

where s_A and s_N are the scattering matrices transforming the wave functions due to Andreev reflection at the interfaces and due to the propagation/scattering in the weak link; the "const." is the phase-independent part of the density of states. The expression $\det\,(1 - s_A s_N)$ equals left-hand side of Eq. 4, where in the continuum, the expressions for $r_A^{\pm}$ have to be properly analytically continued. In the absence of normal reflection, the energy range for the continuum of states formed by decoupled right- and left-movers is $\{E > \Delta + qv_F, E < -\Delta + qv_F\}$ and $\{E > \Delta - qv_F, E < -\Delta - qv_F\}$, respectively. Thus, the current originating from the continuum is

$$I_{\text{cont}} = \frac{2e}{\hbar}\frac{1}{\pi}\text{Im}\left[\int_{\Delta-qv_F}^{\infty} dE\, E\frac{\partial}{\partial E}\frac{d}{d\varphi}\ln\left((r_A^-)^2 - e^{-i\tilde{\varphi}}\right) + \int_{\Delta+qv_F}^{\infty} dE\, E\frac{\partial}{\partial E}\frac{d}{d\varphi}\ln\left((r_A^+)^2 - e^{i\tilde{\varphi}}\right)\right] \quad (10)$$

We evaluate this integral analytically (see Supplementary Materials) and find a phase-independent contribution to the current from the continuum of states

$$I_{\text{cont}} = \frac{2\,eqv_F}{\pi\hbar} \quad (11)$$

This part of the Josephson current is exactly equal to the supercurrent that would flow in an infinite single-mode superconducting wire in the presence of finite Cooper pair momentum q (a detailed discussion can be found in the Supplementary Materials).

Last, the resulting total current through the junction equals

$$I = \frac{e\Delta}{\hbar} = \sin\frac{\tilde{\varphi}}{2}\,\text{sgn}\left(\Delta\cos\frac{\tilde{\varphi}}{2} - qv_F\right) + \frac{2\,eqv_F}{\pi\hbar} \quad (12)$$

It is plotted in Fig. 3A at $qv_F = -0.5\Delta$, shown by the blue line. Because the time-reversal symmetry is broken, the antisymmetry of the current-phase relation $I(\varphi) \neq -I(-\varphi)$ does not hold anymore. In addition, when the phase bias is set to be $\tilde{\varphi} \approx \varphi = 0$, there is still current flowing through the junction. This is a manifestation of the so-called "anomalous" Josephson effect (*17*, *18*, *32*–*35*) occurring in this system.

Because of the identity $I = \frac{2e}{\hbar}\frac{dF}{d\varphi}$, the total current through the junction has to satisfy the condition $\int_0^{2\pi} I(\varphi)\,d\varphi = 0$. Because the current from the bound states is modified in the presence of the Doppler energy shift, now $\int_0^{2\pi} I_{\text{bound}}(\varphi)\,d\varphi \neq 0$. The contribution from the continuum is exactly what makes the condition of the total integral of the current being zero satisfied. Note that in the thermodynamic equilibrium, the free energy of our system is minimized at zero current $I\,|_{\text{eq}} = 0$, which is attained at nonzero phase φ.

Josephson diode effect

The diode effect is quantified by the difference between the magnitudes of the critical currents in the opposite directions $\Delta I_c = I_{c+} - I_{c-}$, which equals

$$\Delta I_c = \text{sgn}(q)\left(\frac{4e\,|\,q\,|\,v_F}{\pi\hbar} - \frac{e\Delta}{\hbar}\left[1 - \sqrt{1 - \left(\frac{qv_F}{\Delta}\right)^2}\,\right]\right) \quad (13)$$

The first term is the nonreciprocal contribution from the continuum of states, and the second term comes from the bound states. The contribution from the continuum of states is phase independent and nonreciprocal, and its sign is determined by the direction of the Cooper pair momentum. The nonreciprocity from the current

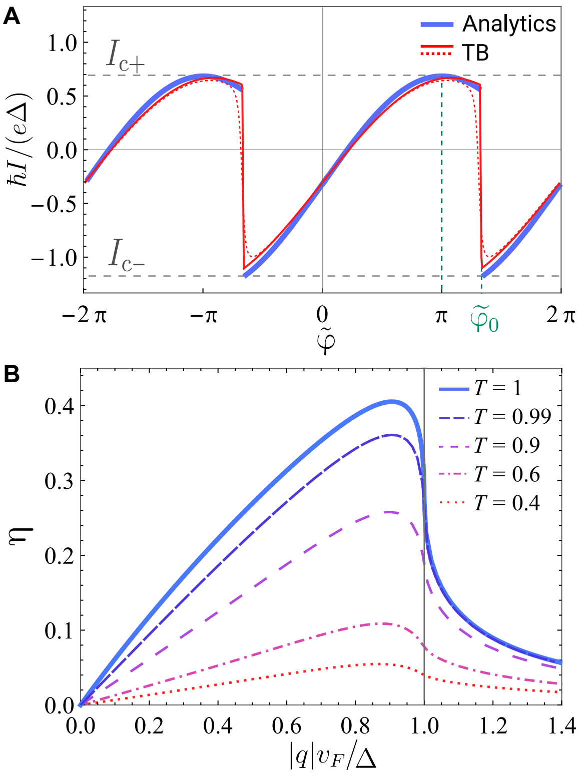

Fig. 3. Current-phase relations and diode efficiency. (**A**) Current-phase relation at $qv_F = -0.5\Delta$. The blue line is the analytical expression (Eq. 12); the red solid and dotted lines correspond to the tight-binding (TB) calculation for transparent junction ($T \approx 1$) and with small normal reflection ($T = 0.99$), respectively. The magnitudes of the critical currents in forward and reverse directions are I_{c+} and I_{c-}, respectively. (**B**) The Josephson diode efficiency $\eta = |\Delta I_c|/(I_{c+} + I_{c-})$ as a function of the Cooper pair momentum q for transparent junction ($T = 1$) and several finite values of junction transparency.

carried by the bound states at $E < 0$ can be understood as follows: The maximum current in the positive direction occurs at $\tilde{\varphi} = \pi$ (as in the case of $q = 0$ Josephson junction), when the bound state is an equal-weight superposition of right-moving electrons and left-moving holes. On the other hand, its partner formed from left-moving electrons and right-moving holes, which would carry the same current in the opposite direction, is inaccessible because it is shifted to $E > 0$ by the Doppler effect. Instead, the largest current in the reverse direction occurs at the branch change $\tilde{\varphi}_0$. The phases corresponding to these points are marked in Figs. 2B and 3A.

The Josephson diode efficiency defined as $\eta \equiv |\Delta I_c|/(I_{c+} + I_{c-})$ is shown in Fig. 3A. $\eta = 1$ corresponds to a perfect supercurrent diode where the critical current is finite in the forward direction and zero in the reverse direction. The maximum diode efficiency that we find reaches about 40%. This corresponds to an asymmetry of $I_{c+}/I_{c-} \approx 230\%$, which is of the order of the largest diode effects reported so far. The maximum efficiency is achieved at a universal point $q_0 v_F = \frac{4\pi}{4+\pi^2}\Delta \approx 0.9\Delta$. Assuming typical values of parameters $\Delta = 0.5$ meV and $\lambda_L = 140$ nm [which are close to those of bulk $NbSe_2$ (*36*)] and $v_F = 10^5$ m/s, we estimate that the magnetic field $B_y \approx 40$ mT is needed to induce optimal Cooper pair momentum $q \sim \Delta/v_F$. This is consistent with the fields used in recent observation of finite Cooper pair momentum and gapless superconductivity in (*27*).

At small q, the diode effect is dominated by the contribution from the continuum of states. Because usually the continuum contribution through the short Josephson junction vanishes, this is a remarkable example where this contribution arises because of time-reversal and inversion symmetry breaking and plays a key role. Note that the first term in Eq. 13 is independent of Δ and the second term becomes large only when q approaches Δ/v_F. Therefore, the asymmetry between the critical currents ΔI_c provides a measure of the Doppler shift in energy, while the critical current itself is the measure of the gap Δ. If one includes the reduction in the gap due to pair-breaking orbital effects or Zeeman field into consideration, then the dominating continuum contribution to the diode efficiency will not change. At the same time, the critical current will decrease, which will lead to an increase in the diode efficiency.

At finite junction transparency T, the condition determining the spectrum of the states in the junction is generalized to

$$T\left((r_A^+)^2 - e^{2iqd+i\varphi}\right)\left((r_A^-)^2 - e^{-2iqd-i\varphi}\right) + \\ +\left(1-T\right)\left[(1 - r_A^- r_A^+)^2 - 4T\right] = 0 \qquad (14)$$

Using this condition and Eq. 9, which can be used to describe bound states and continuum, we compute the current-phase relations and the diode efficiency at finite junction transparency (see section S7A). The results are displayed in Fig. 3B and demonstrate that the effect, although reduced, is robust and persists in the presence of normal reflection.

As recent experiments show (*7*, *37*), it is easy to achieve relatively large values of Cooper pair momentum $qv_F \sim \Delta$. The situation when $|q|v_F > \Delta$ is also possible: It corresponds to gapless superconductivity in the proximitized region (*38*). The gapless superconductivity is possible because the pairing potential in the nanowire is proximity-induced and thus does not have to obey the self-consistency equation. In this case, the presence of mobile quasiparticles at zero energy may complicate the observation of coherent phenomena, such as Josephson effect. We do not address this regime here (the discussion can be found in the Supplementary Materials). For completeness, we show this domain in Fig. 3B as well.

The mechanism for the JDE in short junctions considered in this work is universal because it does not depend on parameters of materials in the junction and its geometry and only relies on finite Cooper pair momentum. This is not the case for long junctions; there, the transport occurring in the normal region becomes important; in particular, a finite-momentum Cooper pair will acquire an additional phase $\delta = 2qd$ as it propagates through the junction. This phase shift is not small in a long junction (*7*), and, additionally, oscillations of the diode efficiency were shown to arise because of the finite junction length. Most of the experiments so far concern the long junction limit, but the microscopic theory of the JDE in long junctions is a matter of future work.

The effect of normal reflection

In the presence of any amount of normal reflection, there are no true bound states in the range of energies $\Delta - |q|v_F < |E| < \Delta + |q|v_F$ anymore. However, at small normal reflection, because of multiple Andreev reflections these states are quasi-bound and, when normal reflection is small, the analytical result above is still relatively accurate. We compare our analytical results for the case of transparent barrier with tight-binding calculation in Fig. 3A without and with a

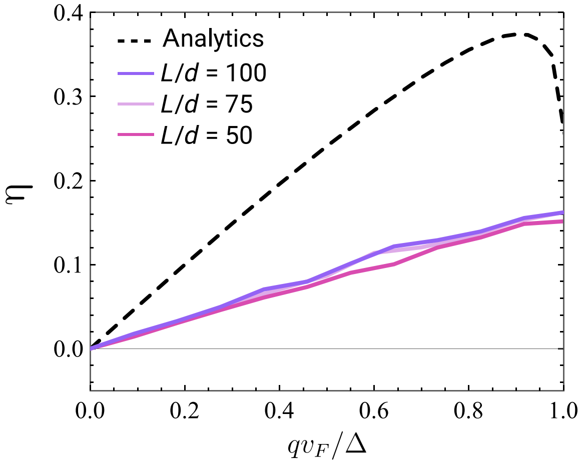

Fig. 4. JDE in the presence of disorder. The results for the diode efficiency from tight-binding simulation are shown at different Cooper pair momenta q (solid lines) averaged over disorder for different lengths of the leads L. The calculations were performed for the system with junction length $d = 4a$ (where a is the lattice spacing) and the length of each lead L. The on-site disorder in chemical potential was uniformly distributed in the range [−10Δ,10Δ], and the results were averaged over 150 realizations. The black dashed line shows the analytical results for a clean system with $T = 0.99$ for comparison.

small barrier at the junction location (see Supplementary Materials for the details of the calculation).

The effect of disorder

We examine the diode effect in the presence of the chemical potential disorder in the system. We performed tight-binding simulations and have found that the diode effect is robust and persists even in the presence of relatively strong disorder. Figure 4 shows the results of the simulations of the Josephson diode in the presence of disorder uniformly distributed in the range [−10Δ,10Δ] for different lengths of the leads. In the calculations, we assumed $\Delta = t/60$, where t is the value of the next-nearest neighbor hopping and performed averaging over 150 realizations of disorder for each system length. As we see from Fig. 4, although the effect is reduced in magnitude, it is robust and does not depend on the system length. We find that the JDE is robust regardless of the ratio between the localization length and the coherence length as long as $d, k_F^{-1} \ll \xi_{\text{loc}}$. Moreover, we find that when $\xi_{\text{loc}} < L$, the sensitivity of the effect to the system length disappears entirely and its magnitude depends on the values of disorder and the superconducting gap only. See the Supplementary Materials for more details and further analysis.

DISCUSSION

Let us discuss other possible ways to realize finite Cooper pair momentum. In Fig. 5, we illustrate a superconductor-ferromagnet bilayer setup, where the magnetic proximity effect from the ferromagnet layer can be used to induce finite-momentum pairing (*10*) and thus achieve the JDE in the absence of external magnetic field. A short junction setup will realize the universal JDE mechanism proposed in this paper, which serves as a clear diagnostic tool for intrinsic finite-momentum pairing. Next, if proximitized system is a topological insulator or a semiconductor with strong spin-orbit coupling, then applying an in-plane magnetic field can lead to finite Cooper pair momentum via the Zeeman effect on spin-helical electrons (*7*, *37*, *38*). Thus, constrictions based on these materials should also be a suitable platform for observing short-junction JDE. After completion of this work, we became aware of a study (*39*) of ϕ_0-Josephson effect in helical edge states of a quantum spin Hall insulator, where a result analogous to Eq. 12 has been found and the presence of critical current asymmetry was recognized. Note that nonmagnetic impurities in the leads and potential barrier in the junction will not induce backscattering in helical edge states of the quantum spin Hall insulator, in contrast to the normal metal or semiconductor wire considered in this work.

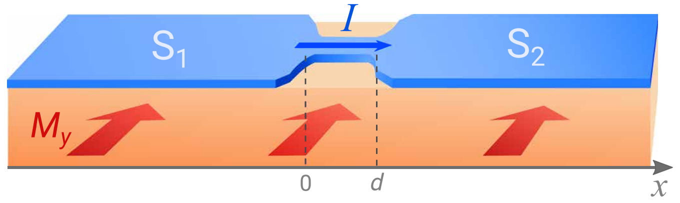

Fig. 5. Proposal for achieving JDE in the absence of external magnetic field. The schematic shows a superconductor with a weak link at $0 < x < d$ that is deposited on top of a magnetic layer magnetized in y direction. The finite Cooper pair momentum is achieved because of the magnetic proximity effect.

In conclusion, we developed a theory for a universal microscopic mechanism for JDE in short Josephson junctions originating from finite Cooper pair momentum q. We found a large asymmetry that reaches 40% when $qv_F \sim \Delta$, which does not depend on the details of the junction. We also proposed a simple way to realize finite-momentum pairing based on the Meissner effect, which makes this mechanism universally applicable.

MATERIALS AND METHODS

The scattering matrix formalism

To obtain the amplitudes of Andreev reflection, we solve the condition on the scattering states $\psi_N^{(1,2)} = s_A|_{x=0,d}\, \psi_S^{(1,2)}$ at each interface ($x = 0$ and $x = d$) using the solutions to the Schrodinger equation in each region. We solve these equations for incoming electrons and holes and obtain the matrix describing Andreev scattering at both interfaces

$$\psi_{\text{out}} = \begin{pmatrix} \psi_{N,e}^{-}(0) \\ \psi_{N,e}^{+}(d) \\ \psi_{N,h}^{+}(0) \\ \psi_{N,h}^{-}(d) \end{pmatrix} = \begin{pmatrix} & & r_A^{-} & 0 \\ & & 0 & r_A^{+} e^{-i\varphi} \\ r_A^{+} & 0 & & \\ 0 & r_A^{-} e^{i\varphi} & & \end{pmatrix} \begin{pmatrix} \psi_{N,e}^{+}(0) \\ \psi_{N,e}^{-}(d) \\ \psi_{N,h}^{-}(0) \\ \psi_{N,h}^{+}(d) \end{pmatrix} \quad (15)$$

$$\equiv s_A^{-1} \psi_{\text{in}}$$

where the unfilled spaces correspond to zero entries and $r_A^{\pm}$ is defined in Eq. 5.

The scattering matrix in the normal region is

$$\psi_{\text{out}} = \begin{pmatrix} \psi_{N,e}^{-}(0) \\ \psi_{N,e}^{+}(d) \\ \psi_{N,h}^{+}(0) \\ \psi_{N,h}^{-}(d) \end{pmatrix} = \begin{pmatrix} r & t' & & \\ t & r' & & \\ & & r^{*} & t'^{*} \\ & & t^{*} & r'^{*} \end{pmatrix} \begin{pmatrix} \psi_{N,e}^{+}(0) \\ \psi_{N,e}^{-}(d) \\ \psi_{N,h}^{-}(0) \\ \psi_{N,h}^{+}(d) \end{pmatrix} \quad (16)$$

$$\equiv s_N \psi_{\text{in}}$$

where in the limit of short junction ($\frac{\Delta d}{v_F} \approx \frac{d}{\xi}, \frac{E_z d}{v_F} \ll 1$) the transmission and reflection coefficients are energy independent.

In the case when there is only one transport channel, $r' = r$ and $t' = t$. The condition determining the spectrum of the bound states is det $(1 - s_N s_A) = 0$, which translates into

$$T\left((r_A^+)^2 - e^{2iqd+i\varphi}\right)\left((r_A^-)^2 - e^{-2iqd-i\varphi}\right)+ \\ +\left(1-T\right)\left[(1-r_A^- r_A^+)^2 - 4T\right] = 0 \tag{17}$$

where $T = |t|^2$, $|t|^2 + |r|^2 = 1$. In the absence of normal reflection $t = e^{iqd}$, $T = 1$ and the condition to Eq. 4.

The current-phase relation

The free energy can be found as (*28*, *29*)

$$F = -\frac{2}{\beta}\sum_{E>0}\ln\left[2\cosh\left(\frac{\beta E}{2}\right)\right] + \int d^2 r \frac{|\Delta|^2}{|g|} + H_0 \tag{18}$$

where H_0 is the particle block of the BdG Hamiltonian and β is the inverse temperature. We neglect the contribution from the spatial integral $\int d^2 r \frac{|\Delta|^2}{|g|}$ to the Josephson current because we assume that Δ changes as a step function at the contacts. Therefore, the free energy can be written as

$$F = -\frac{1}{\beta}\int_0^\infty dE\nu(E)\ln 2\cosh\frac{\beta E}{2} \tag{19}$$

The density of states ν(*E*) in the junction can be evaluated as (*29*)

$$\nu(E) = -\frac{1}{\pi}\mathrm{Im}\frac{\partial}{\partial E}\ln \det\left(1 - s_A s_N\right) + \mathrm{const.} \tag{20}$$

which describes both bound states and the continuum. Here const. term is the phase-independent part of the density of states. We use the expression for the density of states (Eq. 17) together with (Eq. 20) to evaluate the current-phase relation for both transparent and opaque junctions using Eq. 7.

Tight-binding calculations

We simulate the minimal model of short Josephson junction by a nearest-neighbor tight-binding chain with superconducting regions of the same length $L_S = N_S a$ and the thickness of the normal region $L_N = N_N a$, $N_N \ll N_S$. The hopping amplitude *t* is the same in all regions, and the chemical potentials are the same in the superconducting regions (μ_S) and in the normal region (μ_N). The pairing potential at lattice site *n* is $\Delta(n) = \Delta_{1,2} e^{2iqna}$, where $\Delta_1 = \Delta$ and $\Delta_2 = \Delta e^{i\varphi}$. When $t \gg \Delta$, this corresponds to the condition $\mu \gg \Delta$ used in analytical derivations. In all the calculations, we used $\mu_S = 0$, and thus, $v_F = 2at$.

To obtain plots shown in Fig. 3, we used $N_S = 350$, $N_N = 3$ (the total length of the system is 703*a*), $a = 1$, $t = 100$, and $\Delta = 2$. The solid line shows the result at negligible normal reflection, which is achieved at $\mu_N = 0$. The dotted line shows the result at small normal reflection, when a small potential barrier is introduced inside the junction by setting $\mu_N = 25$, which opens a small gap in the dispersion. The current was found by evaluating the expression $I = \frac{2e}{\hbar}\frac{dF}{d\varphi}$ numerically, where the free energy is found by summing over all the negative energy states.

To simulate a system with disorder, we use the parameters $\Delta = 2$, $t = 120$, $a = 1$ for tight-binding calculation and fix the length $N_N = 4$. The disorder is modeled by setting uncorrelated random values of the chemical potential at each lattice site uniformly distributed in a given range. For the figures shown in the main text, the results were averaged over 150 realizations of disorder.

SUPPLEMENTARY MATERIALS

Supplementary material for this article is available at https://science.org/doi/10.1126/sciadv.abo0309

REFERENCES AND NOTES

1. F. Ando, Y. Miyasaka, T. Li, J. Ishizuka, T. Arakawa, Y. Shiota, T. Moriyama, Y. Yanase, T. Ono, Observation of superconducting diode effect. *Nature* **584**, 373–376 (2020).
2. L. Bauriedl, C. Bäuml, L. Fuchs, C. Baumgartner, N. Paulik, J. M. Bauer, K.-Q. Lin, J. M. Lupton, T. Taniguchi, K. Watanabe, C. Strunk, N. Paradiso, Supercurrent diode effect and magnetochiral anisotropy in few-layer $NbSe_2$. arXiv:2110.15752 [cond-mat.supr-con] (29 October 2021).
3. J. Shin, S. Son, J. Yun, G. Park, K. Zhang, Y. J. Shin, J.-G. Park, D. Kim, Magnetic proximity-induced superconducting diode effect and infinite magnetoresistance in van der Waals heterostructure. arXiv:2111.05627 [cond-mat.supr-con] (10 November 2021).
4. E. Bocquillon, R. S. Deacon, J. Wiedenmann, P. Leubner, T. M. Klapwijk, C. Brüne, K. Ishibashi, H. Buhmann, L. W. Molenkamp, Gapless andreev bound states in the quantum spin hall insulator hgte. *Nat. Nanotechnol.* **12**, 137–143 (2017).
5. H. Wu, Y. Wang, P. K. Sivakumar, C. Pasco, U. Filippozzi, S. S. P. Parkin, Y.-J. Zeng, T. McQueen, M. N. Ali, Realization of the field-free Josephson diode. arXiv:2103.15809 (29 March 2021).
6. C. Baumgartner, L. Fuchs, A. Costa, S. Reinhardt, S. Gronin, G. C. Gardner, T. Lindemann, M. J. Manfra, P. E. Faria Junior, D. Kochan, J. Fabian, N. Paradiso, C. Strunk, Supercurrent rectification and magnetochiral effects in symmetric Josephson junctions. *Nat. Nanotechnol.* **17**, 39–44 (2021).
7. B. Pal, A. Chakraborty, P. K. Sivakumar, M. Davydova, A. K. Gopi, A. K. Pandeya, J. A. Krieger, Y. Zhang, M. Date, S. Ju, N. Yuan, N. B. M. Schröter, L. Fu, S. S. P. Parkin, Josephson diode effect from Cooper pair momentum in a topological semimetal. arXiv:2112.11285 [cond-mat.supr-con] (21 December 2021).
8. C. Baumgartner, L. Fuchs, A. Costa, J. P. Cortes, S. Reinhardt, S. Gronin, G. C. Gardner, T. Lindemann, M. J. Manfra, P. E. F. Junior, D. Kochan, J. Fabian, N. Paradiso, C. Strunk, Effect of Rashba and Dresselhaus spin-orbit coupling on supercurrent rectification and magnetochiral anisotropy of ballistic Josephson junctions. arXiv:2111.13983 [cond-mat.supr-con] (27 November 2021).
9. L. P. Gor'kov, E. I. Rashba, Superconducting 2d system with lifted spin degeneracy: Mixed singlet-triplet state. *Phys. Rev. Lett.* **87**, 037004 (2001).
10. N. F. Yuan, L. Fu, Topological metals and finite-momentum superconductors. *Proc. Natl. Acad. Sci.* **118**, e2019063118 (2021).
11. N. F. Q. Yuan, L. Fu, Supercurrent diode effect and finite momentum superconductivity. arXiv:2106.01909 [cond-mat.supr-con] (3 June 2021).
12. A. Daido, Y. Ikeda, Y. Yanase, Intrinsic superconducting diode effect. arXiv:2106.03326 [cond-mat.supr-con] (7 June 2021).
13. J. J. He, Y. Tanaka, N. Nagaosa, A phenomenological theory of superconductor diodes. arXiv:2106.03575 [cond-mat.supr-con] (7 June 2021).
14. H. D. Scammell, J. I. A. Li, M. S. Scheurer, Theory of zero-field diode effect in twisted trilayer graphene. arXiv:2112.09115 [cond-mat.mes-hall] (16 December 2021).
15. S. Ilić, F. S. Bergeret, Theory of the supercurrent diode effect in Rashba superconductors with arbitrary disorder. arXiv:2108.00209 [cond-mat.supr-con] (31 July 2021).
16. N. E. Frattini, U. Vool, S. Shankar, A. Narla, K. M. Sliwa, M. H. Devoret, 3-wave mixing Josephson dipole element. *Appl. Phys. Lett.* **110**, 222603 (2017).
17. T. Yokoyama, M. Eto, Y. V. Nazarov, Josephson current through semiconductor nanowire with spin–orbit interaction in magnetic field. *J. Physical Soc. Jpn* **82**, 054703 (2013).
18. T. Yokoyama, M. Eto, Y. V. Nazarov, Anomalous Josephson effect induced by spin-orbit interaction and zeeman effect in semiconductor nanowires. *Phys. Rev. B* **89**, 195407 (2014).
19. A. Zazunov, R. Egger, T. Jonckheere, T. Martin, Anomalous Josephson current through a spin-orbit coupled quantum dot. *Phys. Rev. Lett.* **103**, 147004 (2009).
20. A. Brunetti, A. Zazunov, A. Kundu, R. Egger, Anomalous Josephson current, incipient time-reversal symmetry breaking, and majorana bound states in interacting multilevel dots. *Phys. Rev. B* **88**, 144515 (2013).
21. A. A. Reynoso, G. Usaj, C. A. Balseiro, D. Feinberg, M. Avignon, Spin-orbit-induced chirality of Andreev states in Josephson junctions. *Phys. Rev. B* **86**, 214519 (2012).
22. C.-Z. Chen, J. J. He, M. N. Ali, G.-H. Lee, K. C. Fong, K. T. Law, Asymmetric Josephson effect in inversion symmetry breaking topological materials. *Phys. Rev. B* **98**, 075430 (2018).
23. S. Pal, C. Benjamin, Quantized Josephson phase battery. *EPL* **126**, 57002 (2019).
24. A. Kopasov, A. Kutlin, A. Mel'nikov, Geometry controlled superconducting diode and anomalous Josephson effect triggered by the topological phase transition in curved proximitized nanowires. *Phys. Rev. B* **103**, 144520 (2021).

25. J. Hu, C. Wu, X. Dai, Proposed design of a Josephson diode. *Phys. Rev. Lett.* **99**, 067004 (2007).
26. Y. Zhang, Y. Gu, J. Hu, K. Jiang, General theory of Josephson diodes. arXiv:2112.08901 [cond-mat.supr-con] (16 December 2021).
27. Z. Zhu, M. Papaj, X.-A. Nie, H.-K. Xu, Y.-S. Gu, X. Yang, D. Guan, S. Wang, Y. Li, C. Liu, J. Luo, Z.-A. Xu, H. Zheng, L. Fu, J.-F. Jia, Discovery of segmented fermi surface induced by cooper pair momentum. *Science* **374**, 1381–1385 (2021).
28. C. W. J. Beenakker, Universal limit of critical-current fluctuations in mesoscopic Josephson junctions. *Phys. Rev. Lett.* **67**, 3836–3839 (1991).
29. C. Beenakker, Three "universal" mesoscopic Josephson effects, in *Transport Phenomena in Mesoscopic Systems* Eds. Hidetoshi Fukuyama, Tsuneya Ando (Springer, 1992) pp. 235–253.
30. A. Furusaki, M. Tsukada, Dc Josephson effect and Andreev reflection. *Solid State Commun.* **78**, 299–302 (1991).
31. P. San-Jose, J. Cayao, E. Prada, R. Aguado, Multiple Andreev reflection and critical current in topological superconducting nanowire junctions. *New J. Phys.* **15**, 075019 (2013).
32. A. Buzdin, Direct coupling between magnetism and superconducting current in the Josephson φ_0 junction. *Phys. Rev. Lett.* **101**, 107005 (2008).
33. A. Assouline, C. Feuillet-Palma, N. Bergeal, T. Zhang, A. Mottaghizadeh, A. Zimmers, E. Lhuillier, M. Eddrie, P. Atkinson, M. Aprili, H. Aubin, Spin-orbit induced phase-shift in Bi_2Se_3 Josephson junctions. *Nat. Commun.* **10**, 126 (2019).
34. W. Mayer, M. C. Dartiailh, J. Yuan, K. S. Wickramasinghe, E. Rossi, J. Shabani, Gate controlled anomalous phase shift in Al/InAs Josephson junctions. *Nat. Commun.* **11**, 212 (2020).
35. E. Strambini, A. Iorio, O. Durante, R. Citro, C. Sanz-Fernandez, C. Guarcello, I. V. Tokatly, A. Braggio, M. Rocci, N. Ligato, V. Zannier, L. Sorba, F. S. Bergeret, F. Giazotto, A Josephson phase battery. *Nat. Nanotechnol.* **15**, 656–660 (2020).
36. J. D. Fletcher, A. Carrington, P. Diener, P. Rodiere, J. P. Brison, R. Prozorov, T. Olheiser, R. W. Giannetta, Penetration depth study of superconducting gap structure of 2*H*–$NbSe_2$. *Phys. Rev. Lett.* **98**, 057003 (2007).
37. S. Hart, H. Ren, M. Kosowsky, G. Ben-Shach, P. Leubner, C. Brune, H. Buhmann, L. W. Molenkamp, B. I. Halperin, A. Yacoby, Controlled finite momentum pairing and spatially varying order parameter in proximitized HgTe quantum wells. *Nat. Phys.* **13**, 87 (2017).
38. N. F. Yuan, L. Fu, Zeeman-induced gapless superconductivity with a partial fermi surface. *Phys. Rev. B* **97**, 115139 (2018).
39. F. Dolcini, M. Houzet, J. S. Meyer, Topological Josephson ϕ_0 junctions. *Phys. Rev. B* **92**, 035428 (2015).

Acknowledgments: We thank N. Yuan, B. Pal, and S. Parkin for related collaborations and A. Ahkmerov for helpful comments on the manuscript. We are grateful for useful discussions with Y. Hou and J. Moodera. **Funding:** This work was supported by the U.S. Army Research Laboratory and the U.S. Army Research Office through the Institute for Soldier anotechnologies, under Collaborative Agreement Number W911NF-18-2-0048. L.F. was partly supported by the David and Lucile Packard Foundation. **Author contributions:** M.D. and L.F. equally contributed to the formulation and analysis of the problem with early contribution by S.P. on the problem formulated for topological-insulator edge states. M.D. performed numerical calculations. M.D. and L.F. equally contributed to the writing of the manuscript with suggestions from S.P. **Competing interests:** The authors declare that they have no competing interests. **Data and materials availability:** All data needed to evaluate the conclusions in the paper are present in the paper and/or the Supplementary Materials.

Submitted 8 January 2022
Accepted 19 April 2022
Published 8 June 2022
10.1126/sciadv.abo0309
