# Supplementary material for: Universal Josephson diode effect
Source: Sci Adv. 2022 Jun 8;8(23):eabo0309. doi: 10.1126/sciadv.abo0309 (PMC9176746; doi:10.1126/sciadv.abo0309)
Supplement: Supplementary file 2 — Sections S1 to S7 Figs. S1 to S12 [file sciadv.abo0309_sm.v2.pdf]

CORRECTED 29 SEPTEMBER 2023; SEE ERRATUM

Science Advances
AAAS

Supplementary Materials for

Universal Josephson diode effect

Margarita Davydova *et al*.

Corresponding author: Margarita Davydova, luthien@mit.edu; Liang Fu, liangfu@mit.edu

Sci. Adv. **8**, eabo0309 (2022)
DOI: 10.1126/sciadv.abo0309

This PDF file includes:

Sections S1 to S7
Figs. S1 to S11

I. SCATTERING STATES

We linearize the problem and consider +/− (where +/− corresponds to the vicinity of $\pm k_F$, i.e. right- and left-movers) and use the ansatz

$$\psi_{1,2} = \begin{pmatrix} a_e^+ e^{ik_F x} e^{i(k+q)x + i\varphi_{1,2}/2} \\ a_h^+ e^{ik_F x} e^{i(k-q)x - i\varphi_{1,2}/2} \\ a_e^- e^{-ik_F x} e^{i(k+q)x + i\varphi_{1,2}/2} \\ a_h^- e^{-ik_F x} e^{i(k-q)x - i\varphi_{1,2}/2} \end{pmatrix} \tag{S1}$$

in superconducting lead 1 and similarly in lead 2. Here $k \equiv k_x$. Recall that $\varphi_1 = 0$ and $\varphi_2 = \varphi$. Notice that normally, there should be wavefunction normalization by the quasiparticle current in order for the scattering problem to be unitary; however, we will use Andreev approximation, which makes this normalization unnecessary.

We assume that $0 < qv_F < \Delta$, and that the energies of the states will lie in the gap of the superconductor $0 < E < \Delta - qv_F$.

The wavefunction for right-movers that decays as $x \to +\infty$ in SC_2:

$$\begin{pmatrix} a_e^+ \\ a_h^+ \\ 0 \\ 0 \end{pmatrix}_{SC_2} = \frac{1}{\sqrt{2}} \begin{pmatrix} \left(\frac{E - v_F q}{\Delta} + i\sqrt{1 - \left(\frac{E - v_F q}{\Delta}\right)^2} \right) \\ 1 \\ 0 \\ 0 \end{pmatrix} \tag{S2}$$

For this state, $v_F k = +i\sqrt{\Delta^2 - (E - v_F q)^2}$.

The L state in SC_1 that decays as $x \to -\infty$ is:

$$\begin{pmatrix} 0 \\ 0 \\ a_e^- \\ a_h^- \end{pmatrix}_{SC_1} = \frac{1}{\sqrt{2}} \begin{pmatrix} 0 \\ 0 \\ \left(\frac{E + v_F q}{\Delta} + i\sqrt{1 - \left(\frac{E + v_F q}{\Delta}\right)^2} \right) \\ 1 \end{pmatrix} \tag{S3}$$

For this state, $v_F k = -i\sqrt{\Delta^2 - (E + v_F q)^2}$.

It is known [29] that the result of the scattering formalism for short junctions will be independent of whether the junction is represented by narrow weak link, a region of normal metal, or an insulating barrier. For simplicity of calculation, we consider normal states in the middle region. We will be solving the problem of Andreev scattering at two interfaces (N/SC_2 and SC_1/N). For incoming electron, the scattering states with that are relevant to the left (1) and the right (2) contacts are:

$$\psi_N^{(1)} = \begin{pmatrix} 0 \\ 0 \\ e^{-ik_F x} e^{ikx} \\ r_A e^{-ik_F x} e^{ikx} \end{pmatrix}, \quad \psi_N^{(2)} = \begin{pmatrix} e^{ik_F x} e^{ikx} \\ r_A e^{ik_F x} e^{ikx} \\ 0 \\ 0 \end{pmatrix} \tag{S4}$$

Where r_A is not necessarily the same constant for scattering on the left and right contacts. For the incoming holes, the problem is set up similarly.

II. THE SCATTERING MATRIX FORMALISM

To obtain the amplitudes of Andreev reflection, we solve the condition $\psi_N^{(1,2)} = S_{interface\ 1,2}\psi_S^{(1,2)}$ at each interface ($x = 0$ and $x = d$). In the case of perfectly transparent contacts, the scattering matrix at the interfaces is identity. We solve these equations (for both incoming electrons and holes at both interfaces) and obtain the matrix describing Andreev scattering at both interfaces:

$$\psi_{out} = \begin{pmatrix} \psi^-_{N,e}(0) \\ \psi^+_{N,e}(d) \\ \psi^+_{N,h}(0) \\ \psi^-_{N,h}(d) \end{pmatrix} = \begin{pmatrix} & & r_A^- & 0 \\ & & 0 & r_A^+ e^{-i\varphi} \\ r_A^+ & 0 & & \\ 0 & r_A^- e^{i\varphi} & & \end{pmatrix} \begin{pmatrix} \psi^+_{N,e}(0) \\ \psi^-_{N,e}(d) \\ \psi^-_{N,h}(0) \\ \psi^+_{N,h}(d) \end{pmatrix} \equiv s_A^{-1}\psi_{in} \tag{S5}$$

where the unfilled spaces correspond to zero entries, and we used the notation

$$r_A^{\pm} = \frac{E \mp v_F q}{\Delta} - i\sqrt{1 - \left(\frac{E \mp v_F q}{\Delta}\right)^2}. \tag{S6}$$

In the absence of normal reflection, the scattering matrix of the normal region is:

$$\psi_{out} = \begin{pmatrix} \psi^-_{N,e}(0) \\ \psi^+_{N,e}(d) \\ \psi^+_{N,h}(0) \\ \psi^-_{N,h}(d) \end{pmatrix} = \begin{pmatrix} r & t' & & \\ t & -r' & & \\ & & r^* & t'^* \\ & & t^* & -r'^* \end{pmatrix} \begin{pmatrix} \psi^+_{N,e}(0) \\ \psi^-_{N,e}(d) \\ \psi^-_{N,h}(0) \\ \psi^+_{N,h}(d) \end{pmatrix} \equiv s_N\psi_{in} \tag{S7}$$

where in the limit of short junction ($\frac{\Delta d}{v_F} \approx \frac{d}{\xi}, \frac{E_z d}{v_F} \ll 1$) the transmission and reflection are energy-independent.

In our case, for one channel, $r' = r$ and $t' = t$. The condition determining the spectrum of the ground states is $\det(\mathbf{1} - s_N s_A) = 0$, which translates into

$$T\left((r_A^+)^2 - e^{2iqd+i\varphi}\right)\left((r_A^-)^2 - e^{-2iqd-i\varphi}\right) + (1-T)\left[(1 - r_A^- r_A^+)^2\right] = 0. \tag{S8}$$

where $T = |t|^2$, $|t|^2 + |r|^2 = 1$. In the absence of normal reflection $t = e^{iqd}$, $T = 1$ and this simplifies to:

$$\left((r_A^+)^2 - e^{2iqd+i\varphi}\right)\left((r_A^-)^2 - e^{-2iqd-i\varphi}\right) = 0 \tag{S9}$$

from the main text, and the solutions to this equation produce the energies of the two bound states (6).

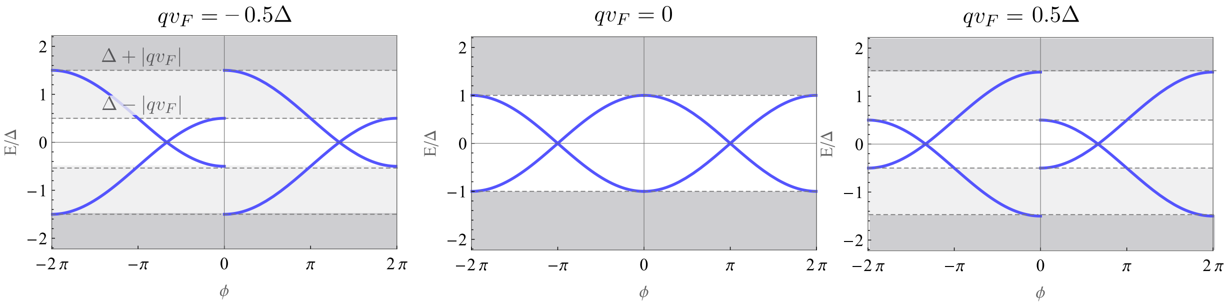

FIG. S1. Spectrum of the bound states in the junction at different values of the Cooper pair momentum q in superconducting regions 1 and 2.

III. THE CURRENT-PHASE RELATION

The free energy is [28, 29]

$$F = -\frac{2}{\beta}\sum_{E>0} \ln\left[2\cosh\left(\frac{\beta E}{2}\right)\right] + \int d^2 r \frac{|\Delta|^2}{|g|} + \mathrm{Tr}\, H_0 \tag{S10}$$

where H_0 is the particle block of the BdG Hamiltonian and β is the inverse temperature. We neglect the contribution from the spatial integral $\int d^2r \frac{|\Delta|^2}{|g|}$ to the Josephson current because we assume that Δ changes as a step-function at the contacts. Therefore, the free energy can be written as

$$F = -\frac{1}{\beta}\int_0^\infty dE \nu(E) \ln 2\cosh\frac{\beta E}{2} \tag{S11}$$

The density of states $\nu(E)$ in the junction can be evaluated as[29],

$$\nu(E) = -\frac{1}{\pi}\,\mathrm{Im}\,\frac{\partial}{\partial E}\ln\det\left(1 - s_A s_N\right) + \mathrm{const.} \tag{S12}$$

which is good for describing both bound and continnum states. Here s_A and s_N are the scattering matrices transforming the wavefunctions due to Andreev reflection at the interfaces and due to the propagation/scattering in the weak link; the 'const.' is the phase-independent part of the density of states.

In the absence of normal reflection, we can rewrite the density of states as

$$\nu(E) = \frac{1}{\pi}\,\mathrm{Im}\,\frac{\partial}{\partial E}\ln\sin\left(\arccos\frac{E+qv_F}{\Delta} + \frac{\widetilde{\varphi}}{2}\right)\sin\left(\arccos\frac{E-qv_F}{\Delta} - \frac{\widetilde{\varphi}}{2}\right) + \mathrm{const.} \tag{S13}$$

where, in order to work with energies of both bound and continuous states, we have to assume that $E = E + i0$, and perform proper analytic continuation where necessary.

Lastly, we plug the expression for the density of states into the free energy and evaluate the current as $I = \frac{2e}{\hbar}\frac{dF}{d\varphi}$. We extend the symmetric integration to $(-\infty, +\infty)$ and integrate by parts using that the boundary terms vanishing as $\propto 1/E$. Thus, we obtain:

$$I(\varphi) = -\frac{e}{2\pi\hbar}\int_{-\infty}^{\infty} dE \tanh\frac{\beta E}{2}\,\mathrm{Im}\frac{\partial}{\partial\varphi}\ln\sin\left(\arccos\frac{E+qv_F}{\Delta} + \frac{\widetilde{\varphi}}{2}\right)\sin\left(\arccos\frac{E-qv_F}{\Delta} - \frac{\widetilde{\varphi}}{2}\right) \tag{S14}$$

which is equal

$$I(\varphi) = -\frac{e}{4\pi\hbar}\int_{-\infty}^{\infty} dE \tanh\frac{\beta E}{2}\,\mathrm{Im}\left[\cot\left(\arccos\frac{E+qv_F}{\Delta} + \frac{\widetilde{\varphi}}{2}\right) - \cot\left(\arccos\frac{E-qv_F}{\Delta} - \frac{\widetilde{\varphi}}{2}\right)\right] \tag{S15}$$

We complete the contour in the upper half complex energy plane, picking up residues at each of the poles of the hyperbolic tangent at Matsubara frequencies $E = i\omega_n \equiv i(2n+1)\pi/\beta$ to obtain a summation:

$$I(\varphi) = \frac{e}{\hbar\beta}\,\mathrm{Re}\left[\cot\left(\arccos\frac{i\omega_n+qv_F}{\Delta} + \frac{\widetilde{\varphi}}{2}\right) - \cot\left(\arccos\frac{i\omega_n-qv_F}{\Delta} - \frac{\widetilde{\varphi}}{2}\right)\right] \tag{S16}$$

Which, finally, can be brought into the form:

$$I(\varphi) = \frac{2e}{\beta\hbar}\sum_{n=0}^{\infty}\mathrm{Re}\cot\left(\arccos\left(\frac{i\omega_n - qv_F}{\Delta}\right) - \frac{\tilde{\varphi}}{2}\right) \tag{S17}$$

The zero temperature result, which we are interested in, is given by turning the sum $\sum_{n=0}^{\infty}$ into an integral $\frac{\beta}{2\pi}\int_0^\infty d\omega$.

1. *The case of* $|q|v_F < \Delta$

Here we derive the Josephson current at zero temperature using a slightly simpler approach than the one introduced in the section above. This approach will also allow us to distinguish the current contributions from bound states and continuous states. The Josephson current can be written as:

$$I = -\frac{2e}{\hbar}\sum_{E>0}\tanh\left(\frac{\beta E}{2}\right)\frac{dE}{d\varphi} - \frac{2e}{\hbar}\frac{2}{\beta}\int_{E\,\in\,\mathrm{cont.}}^{\infty} dE \ln\left[2\cosh\left(\frac{\beta E}{2}\right)\right]\frac{d\nu(E)}{d\varphi} \tag{S18}$$

Where the first term only counts contributions from the bound states and the second term corresponds to the current from the continuum. We are interested in the case when the temperature is zero, and the expression simplifies:

$$I = -\frac{2e}{\hbar}\sum_{E>0}\frac{dE}{d\varphi} - \frac{2e}{\hbar}\int_{E\,\in\,\text{cont.}}^{\infty} dE\, E\frac{d\nu(E)}{d\varphi} \tag{S19}$$

The expression for the contribution from the bound states is given in the main text (eq. (8)). In what follows, we present the details of derivation of the current arising from the continuum of states. $\nu(E)$ equals

$$\nu(E) = -\frac{1}{\pi}\,\text{Im}\,\frac{\partial}{\partial E}\ln\left(T\left((r_A^+)^2 - e^{2iqd+i\varphi}\right)\left((r_A^-)^2 - e^{-2iqd-i\varphi}\right) + (1-T)(1 - r_A^- r_A^+)^2\right) \tag{S20}$$

When $T = 1$, the expression is especially simple. The derivative over φ of the density of states for the continuum of states at $qv_F = 0.5\Delta$ and $\varphi = 0$ is shown in fig. S2.

To compute the current from the continuum of states analytically, we change the order of derivatives and separate the contributions from left- and right-movers:

$$I_{cont} = \frac{2e}{\hbar}\frac{1}{\pi}\,\text{Im}\left[\int_{\Delta+|q|v_F}^{\infty} dE\, E\frac{\partial}{\partial E}\frac{d}{d\varphi}\ln\left((r_A^+)^2 - e^{i\tilde{\varphi}}\right) + \int_{\Delta-|q|v_F}^{\infty} dE\, E\frac{\partial}{\partial E}\frac{d}{d\varphi}\ln\left((r_A^-)^2 - e^{-i\tilde{\varphi}}\right)\right] \tag{S21}$$

Then we proceed to evaluate the derivative over φ:

$$I_{cont} = -\frac{2e}{\hbar}\frac{1}{\pi}\,\text{Im}\, i\left[\int_{\Delta+|q|v_F}^{\infty} dE\, E\frac{\partial}{\partial E}\frac{1}{e^{-i\tilde{\varphi}}(r_A^+)^2 - 1} - \int_{\Delta-|q|v_F}^{\infty} dE\, E\frac{\partial}{\partial E}\frac{1}{e^{i\tilde{\varphi}}(r_A^-)^2 - 1}\right] \tag{S22}$$

Next, we integrate by parts in order to obtain

$$\begin{aligned} I_{cont} = \frac{2e}{\hbar}\frac{1}{\pi}\,\text{Im}\, i\left[(\Delta - |q|v_F)\frac{1}{e^{-i\tilde{\varphi}} - 1} - (\Delta + |q|v_F)\frac{1}{e^{-i\tilde{\varphi}} - 1}\right] + \\ + \frac{2e}{\hbar}\frac{1}{\pi}\,\text{Im}\, i\left[\int_{\Delta+|q|v_F}^{\infty} dE\,\frac{1}{e^{-i\tilde{\varphi}}(r_A^+)^2 - 1} - \int_{\Delta-|q|v_F}^{\infty} dE\,\frac{1}{e^{i\tilde{\varphi}}(r_A^-)^2 - 1}\right] \end{aligned} \tag{S23}$$

Note that $\text{Im}\,\frac{i}{e^{-i\tilde{\varphi}}-1} = \text{Im}\,\frac{i}{e^{i\tilde{\varphi}}-1} = \frac{1}{2}$. Thus, simplifying further:

$$\begin{aligned} I_{cont} &= -\frac{e}{\hbar}\frac{1}{\pi}\left[(\Delta - |q|v_F) - (\Delta + |q|v_F)\right] + \frac{2e\Delta}{\hbar}\frac{1}{\pi}\,\text{Im}\, i\left[\int_1^{\infty} dx\,\frac{1}{e^{-i\tilde{\varphi}+2\text{arccosh}x} - 1} - \int_1^{\infty} dy\,\frac{1}{e^{i\tilde{\varphi}+2\text{arccosh}y} - 1}\right] = \\ &= \frac{e}{\hbar}\frac{2|q|v_F}{\pi} - \frac{2e\Delta}{\hbar}\frac{1}{\pi}\,\text{Im}\left[\int_1^{\infty} dx\,\frac{2e^{2\text{arccosh}x}\sin\tilde{\varphi}}{1 + e^{4\text{arccosh}x} - 2e^{2\text{arccosh}x}\cos\tilde{\varphi}}\right] \end{aligned} \tag{S24}$$

(recall that for continuous states $(r_A^{\pm})^2 = e^{2\text{arccosh}\frac{E\mp qv_F}{\Delta}}$). We see that the imaginary part of the second term is identically zero and thus:

$$I_{cont} = \frac{e\Delta}{\hbar}\frac{2qv_F}{\pi\Delta} \tag{S25}$$

which yields the result in eq. (11).

2. The case $|q|v_F > \Delta$

When $qv_F > \Delta$ and $0 < \tilde{\varphi} < 2\pi$, we evaluate the current using eq. (S17) at zero temperature:

$$I(\varphi) = \frac{e}{\pi\hbar}\int_0^{\infty} d\omega\,\text{Re}\cot\left(\arccos\left(\frac{i\omega - qv_F}{\Delta}\right) - \frac{\tilde{\varphi}}{2}\right) \tag{S26}$$

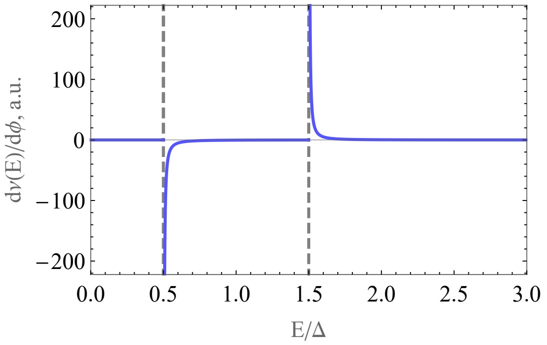

FIG. S2. The derivative over φ of the density of states for the continuum part of the spectrum only at $qv_F = 0.5\Delta$. The dashed gray lines correspond to $E = \Delta - qv_F$ and $E = \Delta + qv_F$. The tails of the quantity $d\nu(E)/d\varphi$ in the continuum have the opposite sign and cancel exactly when $q = 0$. When $q \neq 0$, the energies corresponding to the continuum states for right- and left-movers acquire Doppler shift $\pm qv_F$; on top of that, there is no perfect cancellation anymore, as we show in the calculation above.

We obtain:

$$I(\varphi) = -\frac{2e(qv_F - \sqrt{q^2v_F^2 - \Delta^2})}{\pi} + \frac{2e\Delta\sin\left(\frac{\widetilde{\varphi}}{2}\right)}{2\pi}(\pi - \arg[\Delta + qv_F\cos\left(\frac{\widetilde{\varphi}}{2}\right) + i\sqrt{q^2v_F^2 - \Delta^2}\sin\left(\frac{\widetilde{\varphi}}{2}\right)] \tag{S27}$$

$$-\arg[\Delta - qv_F\cos\left(\frac{\widetilde{\varphi}}{2}\right) + i\sqrt{q^2v_F^2 - \Delta^2}\sin\left(\frac{\widetilde{\varphi}}{2}\right)]) \tag{S28}$$

Which can be simplified to

$$I(\varphi) = -\frac{2e(qv_F - \sqrt{q^2v_F^2 - \Delta^2})}{\pi} + \frac{2e\Delta\sin\left(\frac{\widetilde{\varphi}}{2}\right)}{\pi}\arctan\frac{\Delta\sin\frac{\widetilde{\varphi}}{2}}{\sqrt{q^2v_F^2 - \Delta^2}} \tag{S29}$$

Here $\arg(z)$ refers to the argument of the complex number z.

The maximum negative current occurs at $\widetilde{\varphi} = 0$, $|I_{c-}| = \frac{2e}{\pi}(qv_F - \sqrt{q^2v_F^2 - \Delta^2})$. The maximal positive current occurs at $\widetilde{\varphi} = \pi$, $I_{c+} = \frac{2e}{\pi}(\Delta\sin^{-1}(\Delta/qv_F) - (qv_F - \sqrt{q^2v_F^2 - \Delta^2}))$. Thus, the diode efficiency can be expressed as $\frac{I_{c-} - I_{c+}}{I_{c-} + I_{c+}} = \frac{2(1-\sqrt{1-p^2}) - p\sin^{-1}(p)}{p\sin^{-1}(p)}$, where $p \equiv \Delta/qv_F < 1$.

In the limit $qv_F \gg \Delta$, the diode efficiency approaches $p^2/12 + 13p^4/360 + \ldots$ and thus vanishes as $\propto 1/q^2$. The expression for the supercurrent becomes symmetric in this limit $I(\varphi)|_{qv_F/\Delta\to+\infty} \approx -\frac{e\Delta^2\cos\widetilde{\varphi}}{\pi qv_F}$.

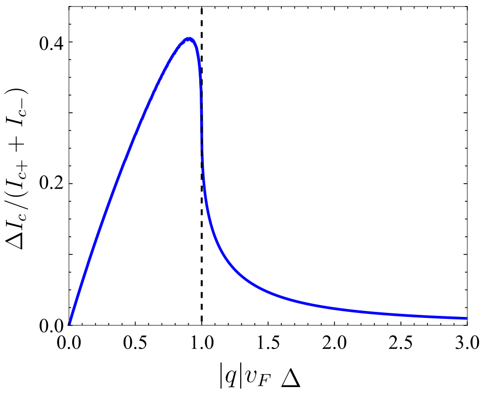

FIG. S3. The diode efficiency η through the junction as a function of the Cooper pair momentum q.

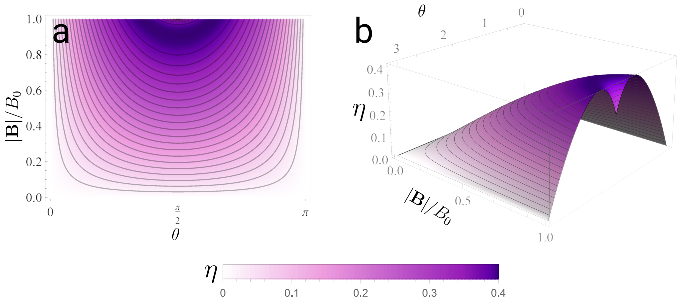

FIG. S4. Dependence of the diode efficiency on the magnitude of magnetic field $|\boldsymbol{B}|$ and the angle θ between the magnetic field and the direction of the current in the domain of parameters satisfying condition $q_x < \Delta/v_F$. B_0 is the value of magnetic field at which $q_x = \Delta/v_F$.

3. *Magnetic field at an angle to the junction*

In a quasi-1D geometry when there are only few transverse modes in the junction, the effect of misaligning the in-plane magnetic field $\boldsymbol{B}$ from the direction y perpendicular to the current is easy to understand. The effect of the x-component of the field, which is in-plane and parallel to the junction leads only to Zeeman energy. At small magnetic fields, this contribution can be neglected, and therefore the Cooper pair momentum in x-direction equals $|\boldsymbol{B}|\sin\theta$, where θ is the angle between the current and direction of the magnetic field. The plot of this dependence is shown in Fig. S4.

IV. TIGHT-BINDING CALCULATIONS

We simulate the minimal model with short Josephson junction by setting up a nearest-neighbor tight-binding chain with superconducting regions of the same length $L_S = N_S a$ and the thickness of the normal region $L_N = N_N a$, $N_N \ll N_S$. The hopping amplitude t is the same in all regions, and the chemical potentials are the same in the superconducting regions μ_S and μ_N in the normal region. The pairing potential at lattice site n is $\Delta(n) = \Delta_{1,2}e^{2iqna}$, where $\Delta_1 = \Delta$ and $\Delta_2 = \Delta e^{i\varphi}$. When $t \gg \Delta$, this corresponds to the condition $\mu \gg \Delta$ used in analytical derivations. In all the calculations, we used $\mu_S = 0$, and thus, $v_F = 2at$.

For calculation in Fig. 3, we used $N_S = 350$, $N_N = 3$ (the total length of the system is $703a$), $a = 1$, $t = 100$ and $\Delta = 2$. The solid line shows the result at negligible normal reflection, which is achieved at $\mu_N = 0$. The dotted line shows the result at small normal reflection, when a small potential barrier is introduced inside the junction by setting $\mu_N = 25$, which opens a small gap in the dispersion, see Fig. S5(a-c).

The current was found by evaluating the expression $I = \frac{2e}{\hbar}\frac{dF}{d\varphi}$ numerically, where the free energy is found by summing over all the negative energy states. We plot it in Fig. Fig. S5(d-f). We estimate that when the potentials at the junction are equal to $\mu_N = 0.1t$ and $\mu_N = 0.4t$, the junction transparency is $T = 0.998$ and $T = 0.975$, respectively. We obtained this correspondence by comparing the energy spectrum obtained from the tight-binding calculation with the analytical expression $E = \sqrt{1 - T\sin^2\frac{\varphi}{2}}$. We estimate the junction transparency to be $T = 0.99$ for the tight-binding calculation at $\mu_N = 0.25t$ shown as a dashed red line in Fig. 3A.

V. SPECTRAL FLOW

In the presence of normal reflection, the left-and right moving states mix, and in the energy domain $\Delta - |q|v_F < |E| < \Delta + |q|v_F$ there are no true bound states anymore. As we see, now the contributions from left-movers and right-movers at these energies (associated with r_A^+ and r_A^-, respectively) are now related. From tight-binding calculations, we see that these states are not connected to the rest of the continuum states as shown in Fig. S6. Upon changing the phase, there is spectral flow of one bound state into these quasi-continuum states and back into another bound states. This allows us to estimate the contribution of the continuum states into the Josephson current based on spectral flow

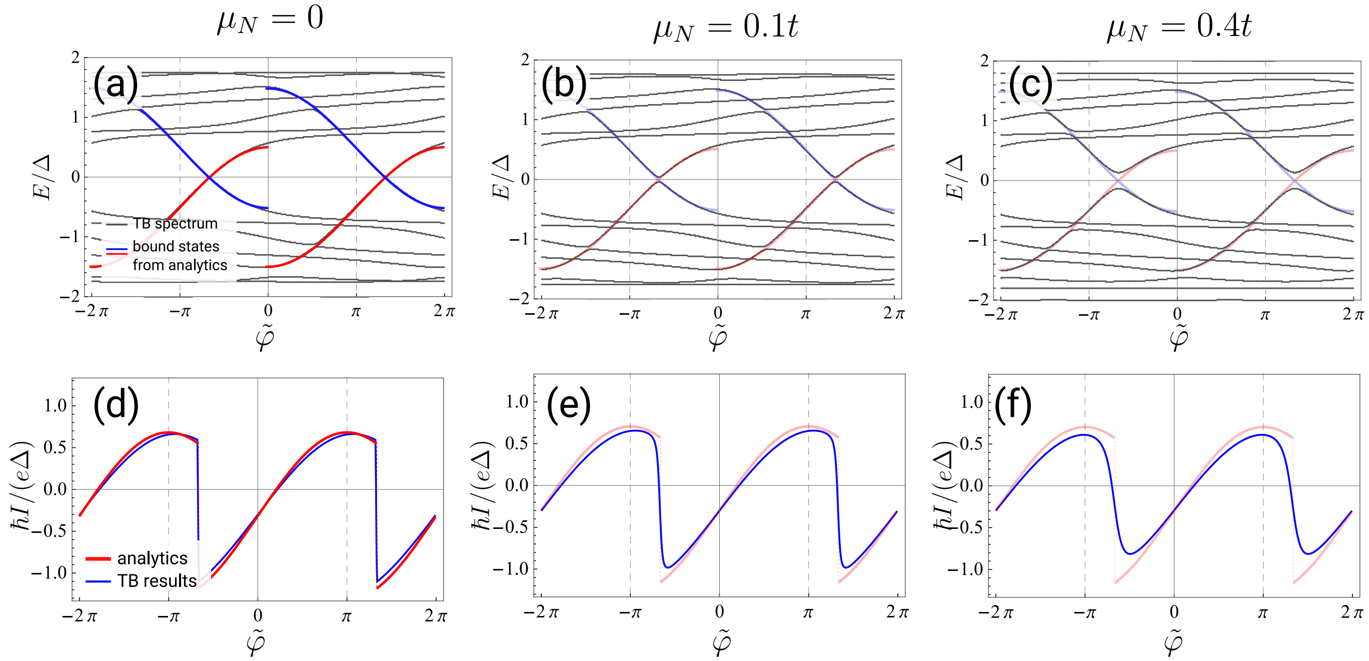

FIG. S5. (a-c) Energy spectra of bound and extended states from tight-binding calculation at $qv_F = -0.5\Delta$. The parameter μ_N is the potential at the junction that controls the amplitude of the normal reflection. The other parameters used are $N_S = 150$, $N_N = 3$, $a = 1$, $t = 20$, $\Delta = 2$. (d-f) Corresponding phase-current relations showing that the current nonreciprocity is decreased when the normal reflection becomes large.

argument:

$$I_{cont} = -\frac{2e}{\hbar}\sum_{i \text{ in continuum}} \frac{d|E_i|}{d\varphi} = \frac{2e}{\hbar}\frac{\Delta E}{\Delta\varphi} = -\frac{2e}{\hbar}\frac{2|q|v_F}{2\pi} = -\frac{e\Delta}{\hbar}\frac{2|q|v_F}{\pi\Delta} \tag{S30}$$

Which exactly matches the result in Eq. (11).

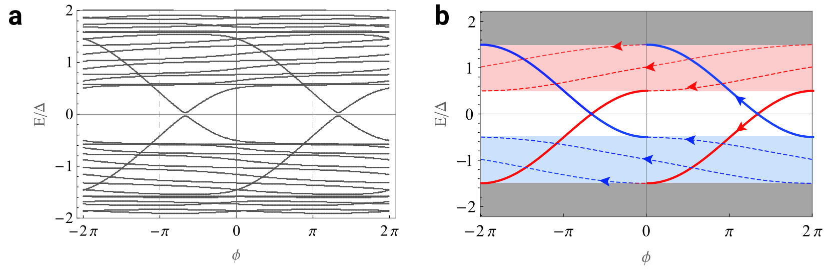

FIG. S6. (a)Tight-binding calculation of the spectrum of a 1D nanowire at $qv_F = -0.5\Delta$. . The parameters used are $N_S = 150$, $N_N = 3$, $a = 1$, $t = 20$, $\Delta = 2$, and $\mu_N = 0$. (b) Analytical expression for bound state levels at $T = 1$ and schematic illustrating the spectral flow connecting the states.

VI. FURTHER DISCUSSION OF THE CONTRIBUTION FROM THE CONTINUUM OF STATES

1. *Vanishing of the contribution from continuum in conventional short junctions*

Let us discuss one perspective on how and why continuous spectrum at $\Delta - |qv_F| < E < \Delta + |qv_F|$ contributes to the Josephson current. As we saw above, the current from continuous states is determined from

$$I_{cont} = -\frac{2e}{\hbar}\int_{\text{cont.}} dE\, E\frac{d\nu(E)}{d\varphi}, \quad \nu(E) = -\frac{1}{\pi}\,\text{Im}\,\frac{\partial}{\partial E}\ln\det\left(1 - s_A s_N\right) + (\varphi\text{-independent const}) \tag{S31}$$

Consider

$$\det\left(1 - s_A s_N\right) = T\left((r_A^-)^2(r_A^+)^2 + 1 - 2\cos\tilde{\varphi}\left[(r_A^-)^2 + (r_A^+)^2\right]\right) - T(1-T)(1-r_A^- r_A^+)^2 - 2i\sin\tilde{\varphi}\left[(r_A^-)^2 - (r_A^+)^2\right] \quad \text{(S32)}$$

In the cases considered in refs.[28–30], the dispersion for left- and right-movers was symmetric and for short junction:

$$r_A^- = r_A^+ = r_A \quad \text{(S33)}$$

Which immediately sets imaginary part of the determinant above zero. Thus, for a short junction with L/R-symmetric dispersion, the density of states is independent of φ and the current from the continuum (S31) vanishes.

It is known that just time-reversal symmetry breaking (for example, induced by spin-splitting magnetic field, see [17, 18]) does not lead to an asymmetry in ABS dispersion in the case of short Josephson junctions. Therefore, the fact that the finite Cooper pair momentum not only breaks time-reversal, but also provides a selected direction in space (inversion breaking) is crucial for the asymmetry and the JDE effect.

2. *Screening current in an infinite superconductor with finite Cooper pair momentum q*

For the rest of the discussion, assume that q is negative, which is the case in Fig. S7. When the energy is in the range $\Delta - |q|v_F < |E| < \Delta + |q|v_F$, the left-moving states in the normal region correspond to a gapless energy range in both superconductors, as seen in Fig. S7.

Let us compute the screening current that flows in an an infinite superconducting slab with $\Delta(x) = \Delta e^{2iqx}$. For the energy range $\Delta - |q|v_F < |E| < \Delta + |q|v_F$ the current comes from left movers only:

$$J_1 = -ev_F \int_{\Delta-|q|v_F}^{\Delta+|q|v_F} \nu_L(E)dE = -\frac{e}{\pi\hbar}\int_{\Delta-|q|v_F}^{\Delta+|q|v_F} \frac{E+|q|v_F}{\sqrt{(E+|q|v_F)^2-\Delta^2}}dE = -\frac{2e}{\pi\hbar}\sqrt{|q|v_F(\Delta+|q|v_F)} \quad \text{(S34)}$$

which, as we see, is zero when $q = 0$. Analogously, the contribution from the true continuum states equals to a difference between the contributions:

$$J_2 = -ev_F\left(\int_{\Delta+|q|v_F}^{\infty}\nu_L(E)dE - \int_{\Delta+|q|v_F}^{\infty}\nu_R(E)dE\right) = \frac{2e}{\pi\hbar}\left(\sqrt{|q|v_F(\Delta+|q|v_F)} - |q|v_F\right) \quad \text{(S35)}$$

Thus, the screening current is

$$J_{scr} = J_1 + J_2 = \frac{2eqv_F}{\pi\hbar}. \quad \text{(S36)}$$

Which, as we see, equals (11).

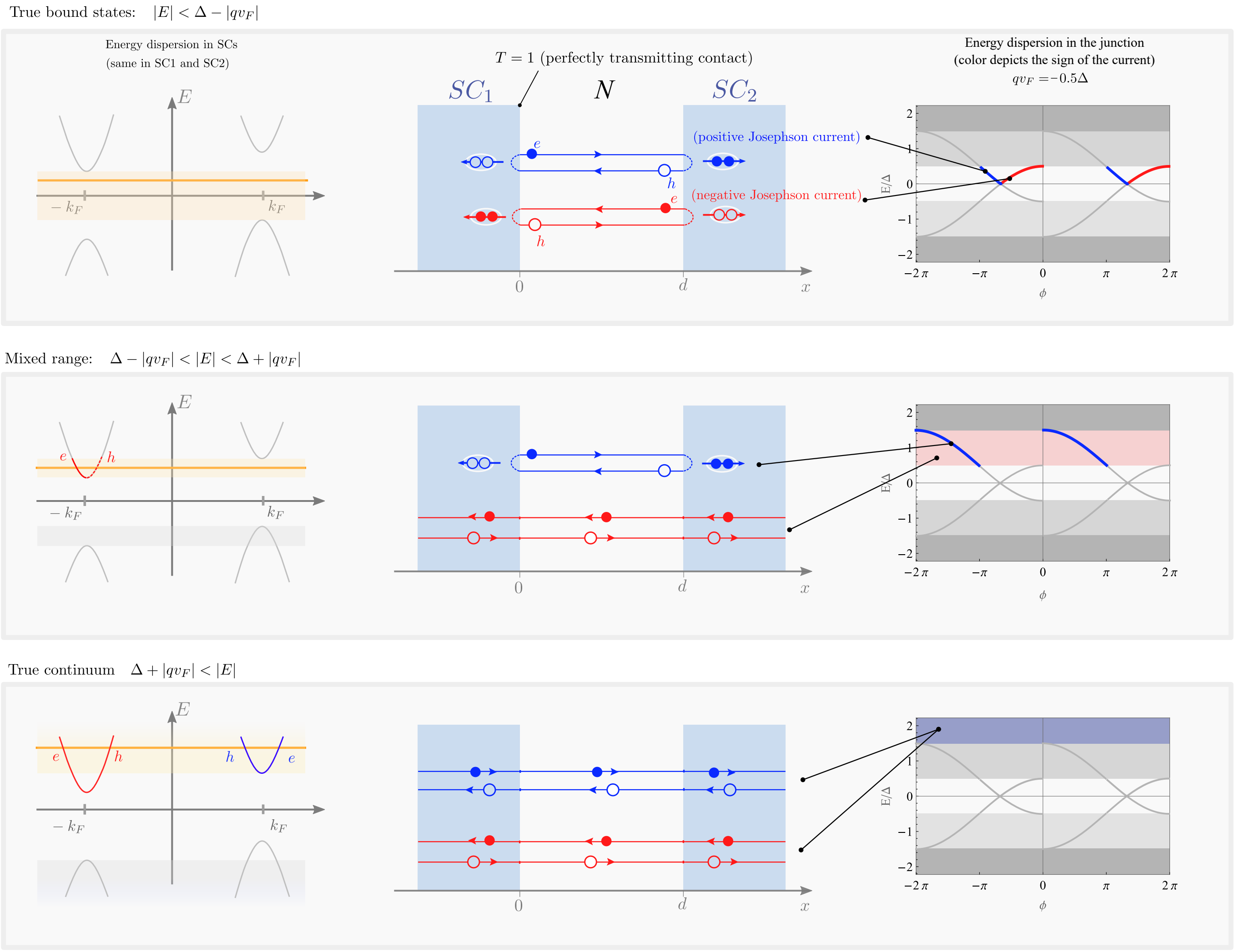

FIG. S7. An illustration showing the types of contributions to the Josephson current at different energy ranges in transparent junction. For simplicity, we only consider positive energy states because the spectrum is particle-hole symmetric. The left column shows the energy spectrum in an exteneded superconductor with finite-momentum pairing $\Delta(x) = \Delta e^{2iqx}$. The second and third columns show the schematics of the states in the junction and the energy spectrum in the junction, respectively. In the energy range $|E| < \Delta - |q|v_F$, there are truly bound Andreev states. In the presence of normal reflection, the bound state in the energy range $\Delta - |q|v_F < E < \Delta + qv_F$ in the middle panel becomes a quasi-bound state. At $|E| > \Delta + qv_F$, the truly continuum states exist, however, they are current-carrying, as we discuss in the text.

VII. EFFECTS OF NORMAL REFLECTION AND DISORDER

A. Effect of normal reflection

Let us derive the expression determining the current-phase relation at finite barrier transparency, i.e. $T < 1$. We use the notation $r_A^{\pm} = e^{i\gamma^{\pm}}$, where $\gamma^{\pm}(E) = \arccos \frac{E \mp qv_F}{\Delta}$ and further simplify (S8):

$$\det(1 - s_A s_N) \propto T \sin\left(\gamma^-(E) + \frac{\widetilde{\varphi}}{2}\right) \sin\left(\gamma^+(E) - \frac{\widetilde{\varphi}}{2}\right) + (1-T)\left[\sin^2\left(\frac{\gamma^-(E)+\gamma^+(E)}{2}\right)\right] = 0 \tag{S37}$$

where, as a reminder $\widetilde{\varphi} = \varphi + 2qd$. We use this expression for evaluation of the density of states as given in eq. (S12) and follow the derivation of the Josephson current through eq. with modified density of states according to eq. (S37). We obtain the expression for the Josephson current through the barrier for a junction with finite transparency at zero temperature:

$$I(\varphi, q) = -\frac{e}{4\pi\hbar}\int d\omega \, \mathrm{Re} \frac{T \sin\left(\gamma^-(i\omega) - \gamma^+(i\omega) + \widetilde{\varphi}\right)}{T \sin\left(\gamma^-(i\omega) + \frac{\widetilde{\varphi}}{2}\right) \sin\left(\gamma^+(i\omega) - \frac{\widetilde{\varphi}}{2}\right) + (1-T)\left[\sin^2\left(\frac{\gamma^-(i\omega)+\gamma^+(i\omega)}{2}\right)\right]} \tag{S38}$$

The current-phase relations computed from this expression at $T < 1$ are shown in Fig. S8. We see that result is similar to the one obtained from tight-binding calculation shown in Fig. S5.

For nearly transparent junction $T \approx 1$, as one can see from, the diode efficiency is non-analytical at $T \approx 1$. Thus, the current cannot be evaluated perturbatively in $(1 - T)$ for nearly transparent junctions.

At the same time, at small transparency $T \ll 1$, one can expand and obtain

$$\begin{aligned} I(\varphi, q) = -\frac{e}{4\pi\hbar}\int d\omega \, \mathrm{Re} \Bigg[&\frac{\sin(\varphi + \gamma^-(i\omega) - \gamma^+(i\omega))}{1 - \cos(\gamma^-(i\omega) + \gamma^+(i\omega))} T + \\ &+ \frac{\sin(\varphi + \gamma^-(i\omega) - \gamma^+(i\omega))}{4\sin^2\left(\frac{\gamma^-(i\omega) - \gamma^+(i\omega)}{2}\right)\left(1 - \cos(\varphi + \gamma^-(i\omega) - \gamma^+(i\omega))\right)} T^2 + O(T^3) \Bigg] \end{aligned} \tag{S39}$$

The leading term $O(T)$ evidently leads to a symmetric current-phase relation and hence, no diode effect. The subleading terms lead to an asymmetric current-phase relation and emergence of the diode effect. As we can see, the diode effect survives at very small junction transparency $T \ll 1$.

B. Effect of disorder

We perform additional tight-binding simulations in order to gain some insight on the effect of disorder.

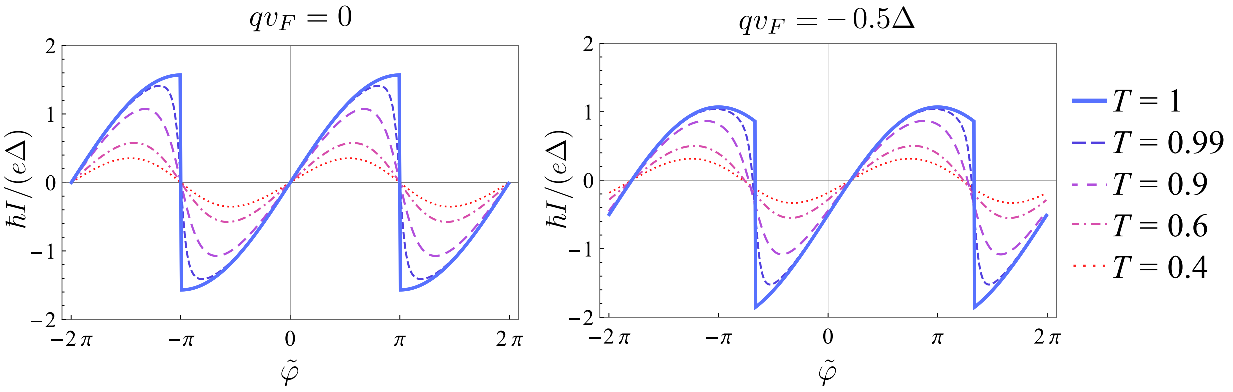

FIG. S8. (a) Current-phase relations at $q = 0$ and $qv_F = -0.5\Delta$ obtained by evaluating expression (S39) at different values of barrier transparency.

We use the parameters $\Delta = 2$, $t = 120$, $a = 1$ in our simulation and fix the length $N_N = 4$. In the figure labels below, we use L and N_S interchangeably.

In Fig. S9A we show the result of the tight-binding simulation for a clean system at different length of the leads $N_S = L = 100, 200, 400$ and 600. We see that the diode efficiency at $L = 400$ and above converges to our analytical result shown by the black dashed line. This panel demonstrates that the effect sensitively depends on the system length when $L/d < 100$, mainly because at this system length the level spacing of the levels in the 'continuum' (that turns into a set of discrete levels because of the finiteness of the system) becomes comparable to Δ.

Fig. S9B shows the diode efficiency averaged over 150 realizations of chemical potential disorder uniformly sampled in the range$[-10\Delta, 10\Delta]$ for $L = 200, 300$ and 400. As we see, at this value of the disorder the diode effect is still present, even though its magnitude is reduced by a factor of two. The effect also becomes system-length independent, which is because at this disorder strength the length L becomes irrelevant in comparison to $\xi_{loc} \ll L$. This has to be contrasted with panel A.

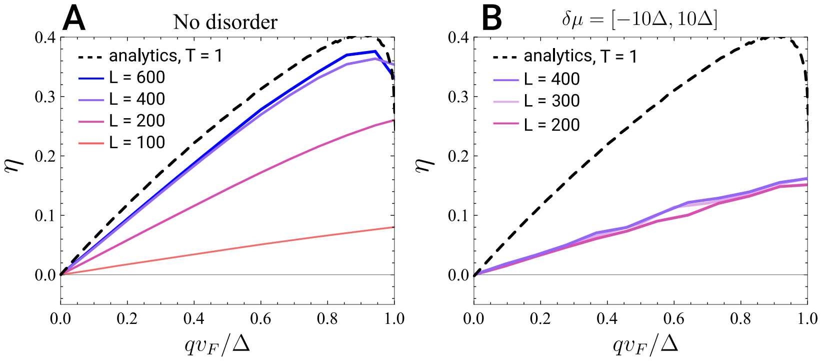

FIG. S9. (A) Tight-binding calculations described in this section for a clean system for different length of the leads. The panel illustrates the sensitivity of the effect on the lead length that appears when $L/d < 100$. (B) Diode efficiency averaged over 150 realizations of chemical potential disorder uniformly sampled in the range$[-10\Delta, 10\Delta]$ for $L = 200, 300$ and 400. The black dashed line is the result obtained from eq.(S39) at $T = 1$ for comparison.

Fig. S10, shows the dependence of the critical currents on the Cooper pair momentum for L = 400. The red lines show the result for the clean system, the black one is the analytical result for the transparent junction for a reference. The blue plots correspond to the critical currents obtained after averaging over 150 realizations of chemical potential disorder uniformly sampled in the range$[-10\Delta, 10\Delta]$. We see that, even though the critical current reduces in value, it is still of the same order as the critical current in a clean system.

In Fig. S11 , the results of the calculations for the clean system are shown in a wider range of the Cooper pair momentum q. At $q > \Delta/v_F$, non-universal oscillations of the effect occur that depend on the system length.

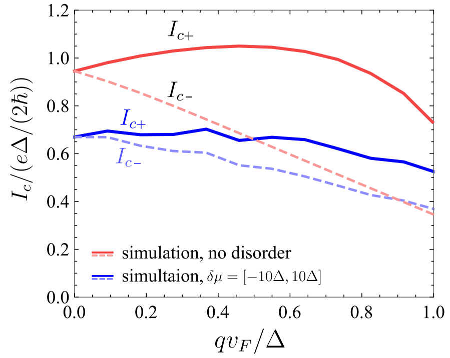

FIG. S10. Dependence of the two critical currents I_{c+} (solid lines) and I_{c-} (dashed lines) on the Cooper pair momentum q. The red plots correspond to the clean system with $L = 400$, the blue ones are averaged over 150 realizations of chemical potential disorder uniformly sampled in the range$[-10\Delta, 10\Delta]$.

We find that when $\xi_{loc} < L$, the sensitivity of the effect to the system length disappears entirely and its magnitude depends on the values of disorder and the superconducting gap only. More importantly, the JDE occurs regardless of

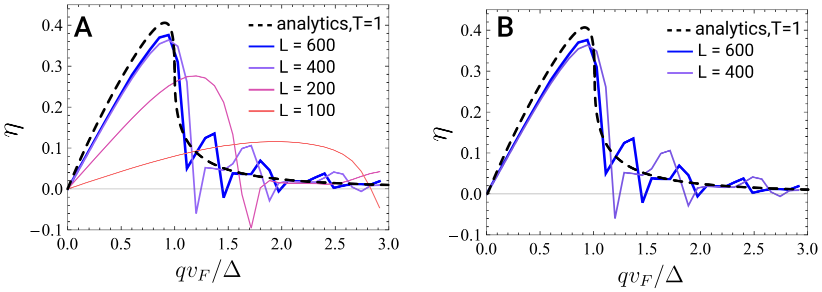

FIG. S11. (A) Tight-binding calculations described in this section for a clean system for different length of the leads in a larger range of Cooper pair momenta. The black dashed line is the result obtained from eq.(S39) at finite junction transparency $T = 1$ for comparison.

the ratio between the localization length and the coherence length as long as $d, k_F^{-1} \ll \xi_{loc}$. This demonstrates that the Josephson diode effect is universally robust.
